# A Novel Homozygous Mutation in *ARL2BP* Causes Multiple Morphological Abnormalities of the Flagella and Primary Ciliary Dyskinesia

**DOI:** 10.1155/humu/7635300

**Published:** 2026-08-25

**Authors:** Ming Li, Wen Tao, Qingshan Ji, Di Yan, Chen Zhang, Qinyi Zhang, Ping Jiang, Jun Qiu, Jie Wen, Bo Xu, Shun Bai, Xiaohua Jiang

**Affiliations:** ^1^ Reproductive Medicine Center, The First Affiliated Hospital of Anhui University of Chinese Medicine, Hefei, Anhui, China, ahtcm.edu.cn; ^2^ School of Medicine, Anhui University of Science and Technology, Huainan, China, aust.edu.cn; ^3^ Department of Ophthalmology, The First Affiliated Hospital of USTC, Division of Life Sciences and Medicine, University of Science and Technology of China, Hefei, China, ustc.edu.cn; ^4^ School of Life Science, Anhui Agricultural University, Hefei, China, ahau.edu.cn; ^5^ Department of Radiology, The First Affiliated Hospital of USTC, Division of Life Sciences and Medicine, University of Science and Technology of China, Hefei, China, ustc.edu.cn; ^6^ Center for Reproduction and Genetics, Department of Obstetrics and Gynecology, The First Affiliated Hospital of USTC, Division of Life Sciences and Medicine, University of Science and Technology of China, Hefei, China, ustc.edu.cn

**Keywords:** *ARL2BP*, male infertility, multiple morphological abnormalities of the flagella, primary ciliary dyskinesia, retinitis pigmentosa

## Abstract

Primary ciliary dyskinesia (PCD) and multiple morphological abnormalities of the sperm flagella (MMAF) frequently co‐occur in male infertility. However, the genetic basis of this syndromic presentation remains unclear. Using whole‐exome sequencing, we identified a novel homozygous *ARL2BP* splice‐site mutation (c.294‐2A>G) in a 23‐year‐old infertile male from a consanguineous family who presented with syndromic PCD and MMAF. This variant causes aberrant pre‐mRNA splicing and triggers nonsense‐mediated mRNA decay, resulting in the complete absence of ARL2BP protein expression. Transmission electron microscopy revealed extensive disorganization of flagellar axonemes with consistent central pair (CP) microtubule depletion and disorganization of peripheral doublets. Immunofluorescence confirmed a severe deficiency of the CP protein SPAG6 in the sperm flagella. Notably, the patient presented without retinal symptoms. Given that *ARL2BP*‐related retinitis pigmentosa generally emerges during the third decade, long‐term ophthalmological follow‐up is essential to detect delayed‐onset retinal degeneration. In conclusion, these findings confirm *ARL2BP* as a causative gene for both PCD and MMAF, expanding the genotypic and phenotypic spectrum of ciliopathies.

## 1. Introduction

Infertility represents a significant global reproductive health burden, affecting 8%–15% of reproductive‐age couples worldwide and carrying significant psychosocial and economic burdens [1, 2]. Male factors contribute to approximately 50% of cases. However, the etiology of male infertility remains unexplained in approximately 30%–40% of cases [3, 4]. Male infertility is a multifactorial disorder characterized by a heterogeneous clinical presentation, including abnormalities in sperm count, motility, and morphology. These are conventionally classified as oligozoospermia, asthenozoospermia, and teratozoospermia, often presenting as a combined oligo‐astheno‐teratozoospermia (OAT) phenotype [5, 6]. Among these, asthenozoospermia is the most frequent isolated semen abnormality, identified in 19% infertile men, and is present in up to 63% when combined with oligozoospermia or teratozoospermia [7, 8].

Primary ciliary dyskinesia (PCD) is an autosomal recessive disorder of motile cilia characterized by chronic respiratory disease, situs inversus, and infertility [9–12]. Pathogenic variants in more than 50 genes encoding axonemal dyneins, radial spokes, nexin–dynein regulatory complexes (N‐DRC), or cytoplasmic assembly factors have been implicated in PCD [12–14]. Respiratory motile cilia and sperm flagella share a highly conserved 9 + 2 axonemal architecture, consisting of nine peripheral doublet microtubules surrounding a CP of singlet microtubules. Consequently, mutations causing PCD frequently impair sperm motility and result in male infertility [15–18].

Multiple morphological abnormalities of the flagella (MMAF) represent one of the most severe forms of asthenozoospermia and is defined by the combination of absent, short, bent, coiled, or irregular flagella [19–21]. Whole‐exome sequencing (WES) has enabled the identification of multiple gene families strongly associated with the MMAF phenotype, including the dynein axonemal heavy chain (DNAH) family [22–26], the coiled‐coil domain‐containing (CCDC) protein family [22, 27–30] and the cilia‐ and flagella‐associated protein (CFAP) family [19, 22, 31–36]. Despite the established genetic basis of MMAF, the underlying causes remain unidentified in a substantial proportion of cases.

ARL2BP (ADP‐ribosylation factor‐like 2 binding protein) localizes to the basal body, transition zone, and axoneme of photoreceptor connecting cilia, where it stabilizes doublet microtubules and maintains intraflagellar transport [37–39]. Global *Arl2bp* knockout in mice displays retinal degeneration, impaired spermatogenesis, situs inversus, and enlarged brain ventricles [40]. Since Davidson et al. [37] first linked *ARL2BP* to autosomal recessive retinitis pigmentosa (RP), the associated phenotypic spectrum has progressively expanded. Moye et al. [40] initially established the association between *ARL2BP* mutations and male infertility, reporting two consanguineous Portuguese families carrying homozygous variants (c.207+1G>A and c.33_36delGTCT) in which affected individuals presented with RP and severe asthenozoospermia. The patient sperm have shortened and misassembled tails, loss of axonemal doublets, and complete immotility, and the *Arl2bp*‐knockout mouse model recapitulated the human phenotypes [40]. Zhu et al. [41] identified a Chinese patient with the variant c.22_23delAG presenting with the classic triad of RP, complete situs inversus, and oligozoospermia. More recently, Placidi et al. [42] reported a case with a splice‐site variant (c.294‐1G>C) causing rod‐cone dystrophy, situs inversus, asthenozoospermia, and unilateral renal agenesis with microcysts, further expanding the phenotypic spectrum.

In this study, WES of an infertile male with MMAF and PCD from a consanguineous family identified a novel homozygous splice‐site mutation (c.294‐2A>G) in *ARL2BP*. This mutation leads to *ARL2BP* loss‐of‐function, resulting in disorganization of the axonemal structure and reduced sperm motility.

## 2. Materials and Methods

### 2.1. Participants

A 23‐year‐old infertile patient with PCD from a Chinese consanguineous family was recruited for this study. Peripheral venous blood was collected from the patient and his family members for genetic analysis. This study was approved by the Ethics Committee of the First Affiliated Hospital of the University of Science and Technology of China (2024AH‐36‐01). Written informed consent was obtained from all participants in accordance with the Declaration of Helsinki and institutional ethical guidelines.

### 2.2. Semen Parameters and Sperm Morphological Analysis

Following a period of sexual abstinence from 2 to 7 days, semen samples were obtained via masturbation. Semen volume was calculated by weighting according to World Health Organization (WHO) guidelines [43]. Sperm concentration and motility were evaluated using computer‐assisted sperm analysis (CASA, SAS‐II, Beijing, China). Sperm morphology was assessed via Diff‐Quick staining (Ankebio, Hefei, China). At least 200 spermatozoa per sample were examined under a light microscope (UB100i, UOP, Chongqing, China) at 100× magnification [43]. Structural defects, including flagellar abnormalities, were recorded and quantified.

### 2.3. WES and variant filtration

WES was performed on the affected individual as previously described [32, 44]. Genomic DNA was extracted from peripheral blood samples using the QIAamp DNA Blood Mini Kit (QIAGEN, Shanghai, China) according to the manufacturer′s protocol. Exome capture was carried out using the Agilent SureSelect Human All Exon Kit. Libraries were then prepared and sequenced on Illumina NextSeq 500 and NovaSeq 6000 (Illumina, San Diego, California, United States). The Burrows‐Wheeler Aligner (BWA) was subsequently used to align the FASTQ reads to the GRCh38/hg38 human reference genome [45].

Variant filtering was performed as previously described with minor modifications [46]. In detail, filtering criteria included: (1) inclusion of autosomal recessive variants, given the patient′s consanguineous family background; (2) inclusion of variants located within linkage regions with logarithm of the odds (LOD) scores > 0.58; (3) inclusion of protein‐altering variants, including frameshift, splicing, stop‐gain, and nonsynonymous variants; (4) exclusion of variants with a minor allele frequency (MAF) > 0.01 in the 1000 Genomes, ESP6500, ExAC, or gnomAD databases; (5) exclusion of variants that were predicted to be nondeleterious by > 50% of in silico prediction tools; (6) exclusion of variants in genes with no established role in spermatogenesis, based on knockout mouse phenotypes recorded by the Mouse Genome Informatics (MGI) database [47]; and (7) inclusion of variants in genes expressed in testis. Sanger sequencing was performed on the patient and his parents to validate candidate variants.

### 2.4. PCR and Real‐Time RT‐PCR

Sperm were collected by centrifugation and lysed with a solution containing 0.05% SDS, 1% Triton X‐100, and 1% DEPC water. RNA extraction and RT‐PCR were performed as previously described [46]. The sequences of the primers used were as follows: *ARL2BP*, forward 5 ^′^‐ACCTGGAGTTTGAAGACACAG‐3 ^′^ and reverse 5 ^′^‐AGTCACCACTAAGCCACTGC‐3 ^′^; *ACTIN*, forward 5 ^′^‐AATGAGCTGCGTGTGGCTC‐3 ^′^ and reverse 5 ^′^‐ATAGCACAGCCTGGATAGCAAC‐3 ^′^.

### 2.5. Western Blot

Sperm were lysed in ice‐cold RIPA buffer supplemented with phosphatase and protease inhibitors [48]. A total of 20 *μ*g of protein was loaded on a 12% SDS‐PAGE gel and subsequently transferred onto an Immobilon‐P membrane (IPVH00010; Millipore) with a pore size of 0.45 *μ*m. The membrane was blocked in TBST buffer (50 mM Tris, pH 7.4, 150 mM NaCl, and 0.1% Tween‐20) containing 5% nonfat milk for 1 h at room temperature. Primary antibody incubation proceeded overnight at 4°C with anti‐ARL2BP (1:1000; 10090‐2‐AP; Proteintech, China) and anti‐GAPDH (1:10,000; 60004‐1‐Ig; Proteintech, China). After washing with TBST, the membrane was further incubated with horseradish peroxidase (HRP)–conjugated secondary antibodies (1:5000, HRP donkey antirabbit IgG, 406401, BioLegend, United States; 1:5000, HRP goat antimouse IgG, 405306, BioLegend, United States) at room temperature. Immunoreactive bands were visualized by enhanced chemiluminescence.

### 2.6. Transmission Electron Microscopy (TEM) Analysis

Semen samples from the patient were centrifuged at 400 × g for 3 min at room temperature. After washing in PBS, the pellets were fixed overnight in 2.5% glutaraldehyde at 4°C. Following four rinses with 0.1‐M phosphate buffer, the samples were subjected to postfixation in 1% osmium tetroxide (OsO_4_). The samples were then dehydrated through a graded ethanol series, infiltrated with acetone, and embedded in epoxy resin. Ultrathin sections were cut using an ultramicrotome and examined by TEM. The sections were stained with lead citrate and uranyl acetate. TEM analysis of spermatozoa from the patient and control individuals was performed as described previously [49].

### 2.7. Immunofluorescence (IF) Staining

Sperm samples were fixed in 4% paraformaldehyde for 30 min at 4°C, permeabilized with 0.3% Triton X‐100, and washed with PBS. After blocking with 5% bovine serum albumin (BSA) in PBS for 60 min at room temperature, samples were incubated with primary antibodies overnight at 4°C, followed by secondary antibodies for 1 h at 37°C. The following primary antibodies and secondary antibodies were used: anti‐DNAH3 (1:200; ab172632; Abcam, United States), anti–*β*‐tubulin (1:200; 66240‐1‐Ig; Proteintech, China), anti‐SPAG6 (1:200; D260971; Proteintech, China), anti‐ODF2 (1:200; 12058‐1; Sigma, United States), anti‐AKAP3 (1:200; 13907‐1‐AP; Proteintech, China), Alexa Fluor 488 (1:200; SA00013‐1; Thermo, United States), and Alexa Fluor 594 (1:200; SA00013‐4; Thermo, United States). Nuclei were counterstained with DAPI (1:500; C0060; Solarbio, China) for 15 min at 37°C

## 3. Results

### 3.1. Clinical Characteristics and Semen Parameters of the Infertile Patient

**Figure 1:**
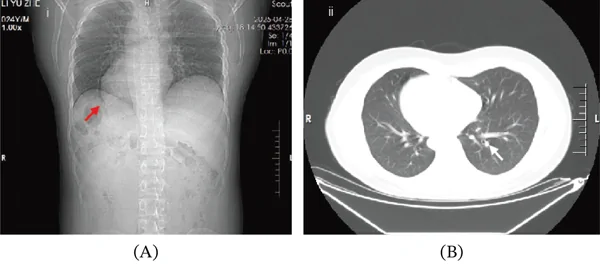
Radiological examination of the proband (IV:1). (A) The x‐ray (i) reveals typical dextrocardia (indicated by the red arrow). (B) The chest high‐resolution computed tomography (HRCT) (ii) demonstrates multiple cystic bronchiectasis, thickening of the bronchial wall, and signs of infection, manifested as multiple spot‐ and sheet‐like areas of increased density in both lung lobes (indicated by the white arrow).

**Figure 2:**
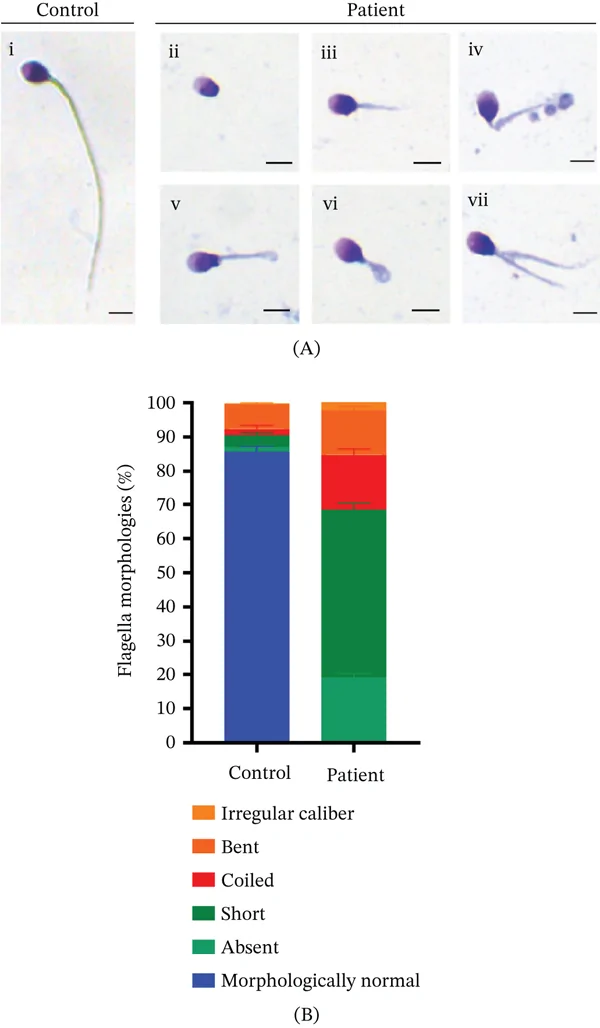
The patient with *ARL2BP* mutation exhibited a diverse range of abnormalities in sperm morphology. (A) Diff‐Quik staining revealed sperm morphological abnormalities in the patient, including absent (ii), short (iii), bent (iv), coiled (v, vi), or irregular (vii) sperm flagella (scale bars: 5 *μ*m). (B) The proportions of sperm flagellar abnormalities are presented as the mean ± SD. Three independent experiments were performed, and at least 200 sperm were examined for each time per individual.

We recruited an infertile male from a consanguineous family who had been unable to conceive after at least 2 years of marriage. He had no history of smoking, alcohol consumption, or exposure to occupational toxicants. Physical examination revealed normal height, weight, external genitalia, and testicular volume. Chromosomal analysis showed a normal 46, XY karyotype, and no Y chromosome microdeletions were detected. Notably, chest radiography and computed tomography revealed dextrocardia (Figure 1, red arrow) and bilateral pulmonary abnormalities, including cystic dilatation and infectious changes characterized by multifocal spot‐like and patchy opacities (Figure 1, white arrow), suggestive of PCD. Semen analysis showed normal ejaculate volume, but sperm concentration was reduced to 4.32 ± 0.89 × 10^6^/mL, with a total count of 12.28 ± 4.76 × 10^6^ per ejaculate. All spermatozoa displayed severely impaired progressive motility and flagellar morphological abnormalities (Table 1). To further assess sperm morphology, Diff‐Quik staining was performed on spermatozoa from fertile controls and the patient (Figure 2A). The patient′s spermatozoa exhibited a high frequency of flagellar abnormalities, including short (52.84*%* ± 1.84*%*), coiled (12.30*%* ± 2.89*%*), bent (8.00*%* ± 2.89*%*), absent (24.79*%* ± 3.62*%*), and irregular (2.07*%* ± 0.27*%*) (Figure 2B). Collectively, these findings are consistent with a diagnosis of MMAF.

**Table 1:** Clinical characteristics of patient′s sperm.

	Reference values	I	II	III	*M* *e* *a* *n* ± *S* *D*
Semen parameters:					
Semen volume (mL)	≥ 1.5	3.0	3.2	2.1	2.77 ± 0.58
Sperm concentration (10^6^/mL)	≥ 15	4.47	5.12	3.36	4.32 ± 0.89
Sperm count (10^6^)	≥ 39	13.41	16.38	7.06	12.28 ± 4.76
Semen pH	Alkaline	Alkaline	Alkaline	Alkaline	Alkaline
Motile sperm (%)	≥ 40	0	0	0	0
Progressively motile sperm (%)	≥ 32	0	0	0	0
Immobile (IM) sperm (%)		100	100	100	100
Sperm morphology analysis:					
Tail defective sperm rate (%)		100	100	100	100
Absent (%)		28.17	25.24	20.98	24.79 ± 3.62
Short (%)		54.46	53.81	50.24	52.84 ± 1.84
Coiled (%)		9.86	11.43	15.61	12.30 ± 2.89
Bent (%)		5.68	7.14	11.22	8.00 ± 2.89
Irregular caliber (%)		1.88	2.38	1.95	2.07 ± 0.27
Normal morphology sperm rate (%)	≥ 4	0	0	0	0
Abnormality sperm rate (%)		100	100	100	100

*Note:* Data from three semen analyses (I–III) are presented as mean ± SD. Reference values are based on the WHO fifth edition guidelines. The criterion for collecting ejaculate Samples I, II, and III is a period of sexual abstinence within 2–7 days. Semen parameters included the total amount of ejaculate at one time, and at least 200 spermatozoa were assessed for morphology in each sample.

### 3.2. Identification of a Pathogenic Splice‐Site Mutation in *ARL2BP*

**Figure 3:**
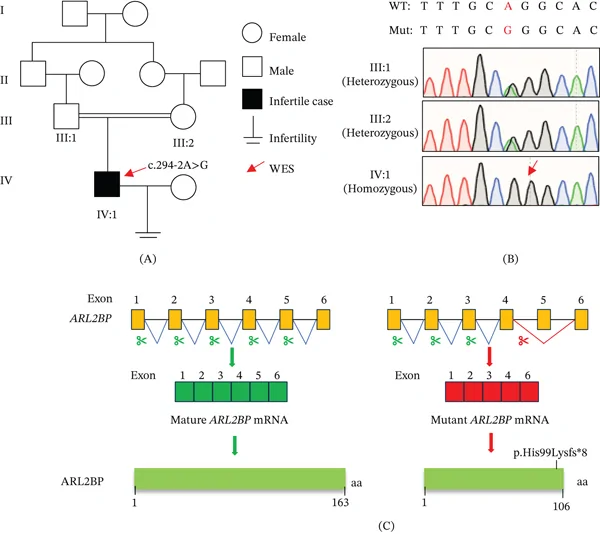
Identification of a homozygous *ARL2BP* variant in an infertile man born to first‐cousin parents. (A) Pedigree of the studied family. Squares denote males, whereas circles represent females. Solid symbols signify affected individuals, and open symbols represent unaffected individuals. Double horizontal lines indicate consanguineous unions. Slashes mark deceased members. Red arrows point to individuals selected for whole‐exome sequencing. (B) Sanger sequencing chromatograms of the identified variant (c.294‐2A>G); the arrow indicates its location. (C) Schematic representation of normal and aberrant splicing patterns of *ARL2BP*. The c.294‐2A>G mutation (GRCh38, ENST00000219204.8) is predicted to disrupt the canonical acceptor splice site at the Intron 4 to Exon 5 junction, leading to specific aberrant splicing, such as Exon 5 skipping or activation of an alternative splice site.

Given that the patient was born to consanguineous parents (Figure 3A), WES and bioinformatic analyses were performed to investigate the genetic basis of MMAF. A homozygous splice‐site mutation affecting the canonical acceptor site (GRCh38, ENST00000219204.8, c.294‐2A>G) of *ARL2BP* was identified (Figure 3A). Sanger sequencing confirmed the mutation, revealing homozygosity in the patient and heterozygosity in his parents (Figure 3B). This finding is consistent with an autosomal recessive mode of inheritance.

To evaluate the functional effect of the variant on splicing, in silico prediction analyses were performed using Human Splicing Finder and MaxEntScan [50]. Both algorithms consistently predicted that the c.294‐2A>G variant disrupts the canonical acceptor splice site at the Intron 4 to Exon 5 junction [50]. The variant is predicted to impair normal mRNA processing, leading to Exon 5 skipping or aberrant splicing, which would ultimately result in protein truncation (p.His99Lysfs∗8) and loss of ARL2BP function (Figure 3B).

### 3.3. Impact of the *ARL2BP* Mutation on Transcript and Protein Expression

**Figure 4:**
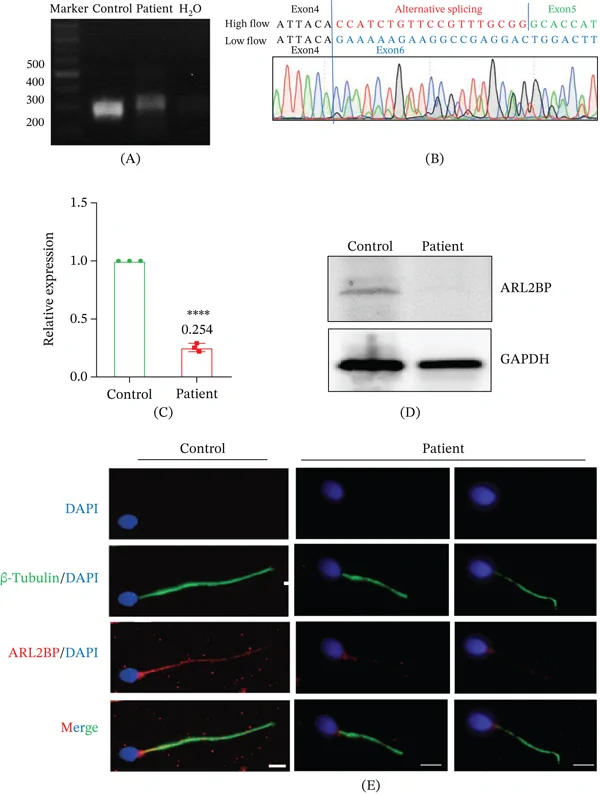
Effect of the *ARL2BP* variant on gene expression. (A) Agarose gel electrophoresis of PCR‐amplified ARL2BP cDNA fragments encompassing Exons 3–6, derived from spermatozoa‐derived RNA. (B) Sanger sequencing chromatogram of the PCR product from the patient presented in Panel A. Sanger sequencing indicated that the c.294‐2A>G mutation disrupted the canonical splice acceptor site, resulting in two aberrant splicing products. Exon 4 sequences are depicted in black, Exon 5 sequences in green, and Exon 6 sequences in blue. Intron 4 sequences retained via alternative splicing are highlighted in red. (C) Quantitative real‐time PCR analysis of *ARL2BP* mRNA expression in human spermatozoa from a normal control and the patient. *ACTB* was used as an internal control. (D) Western blot analysis of ARL2BP protein levels in sperm from a normal control and the patient. ARL2BP protein expression was undetected in the patient′s sperm. (E) Immunofluorescence staining of ARL2BP in human spermatozoa from a normal control and the patient. DAPI (blue), *β*‐tubulin (green), and ARL2BP (red). Scale bars: 5 *μ*m. For Panels C–E, three independent experiments were performed.

Splice‐site mutations that introduce premature termination codons often trigger nonsense‐mediated mRNA decay [51]. To assess this possibility, the expression of *ARL2BP* mRNA in sperm was evaluated via RT‐PCR. Agarose gel electrophoresis of the PCR products revealed a single band of the expected size (293 bp) in the control sample. In contrast, the patient′s sample exhibited a band of higher molecular weight with diminished intensity (Figure 4A). Sanger sequencing verified that the c.294‐2A>G variant disrupts normal pre‐mRNA splicing, resulting in multiple abnormal transcripts: (i) the insertion of 19 base pairs from the 3 ^′^ end of Intron 4 at the Intron 4 to Exon 5 junction and (ii) the complete skipping of Exon 5. Both transcripts are predicted to encode truncated proteins. These findings provide functional evidence validating the pathogenicity of this splice‐site mutation (Figure 4B). Quantitative real‐time PCR results indicated that the residual level of ARL2BP mRNA in the patient was approximately 25% of that in the control group (Figure 4C), which implies the instability of the mutant transcripts. Western blot analysis demonstrated the complete absence of the ARL2BP protein in the patient′s spermatozoa (Figure 4D), consistent with reduced mRNA expression. IF staining showed that ARL2BP was localized along the entire flagellum in the control sperm, with normal axonemes marked by *β*‐tubulin staining (Figure 4E). In the spermatozoa of the patient, only faint, presumably nonspecific staining was observed. Collectively, these findings indicate that the *ARL2BP* splice mutation leads to the complete loss of protein expression.

### 3.4. Disruption of Axonemal Structure in Patient Spermatozoa

**Figure 5:**
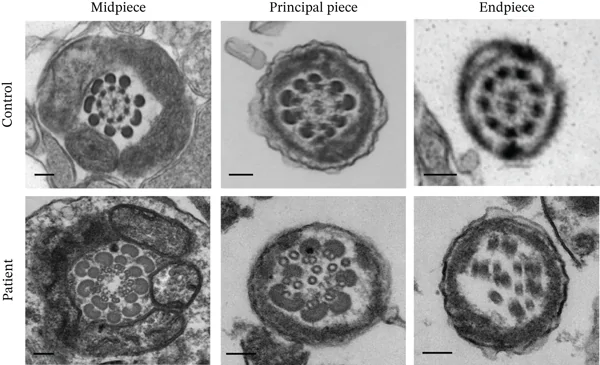
Ultrastructural flagellar abnormalities in the patient. Representative TEM micrographs showing cross sections of the midpiece, principal piece, and end piece of sperm flagella from a fertile control and the patient. Scale bars represent 100 nm.

To explore the mechanism underlying the sperm defects in the patient, the ultrastructure of mature sperm flagella was examined TEM. In comparison with the typical “9 + 2” axonemal arrangement observed in the fertile control, the majority of flagella from the patient presented severely disorganized axonemes, with loss of peripheral microtubule doublets and/or the CP. Additionally, supernumerary outer dense fibers (ODFs) were frequently observed (Figure 5).

**Figure 6:**
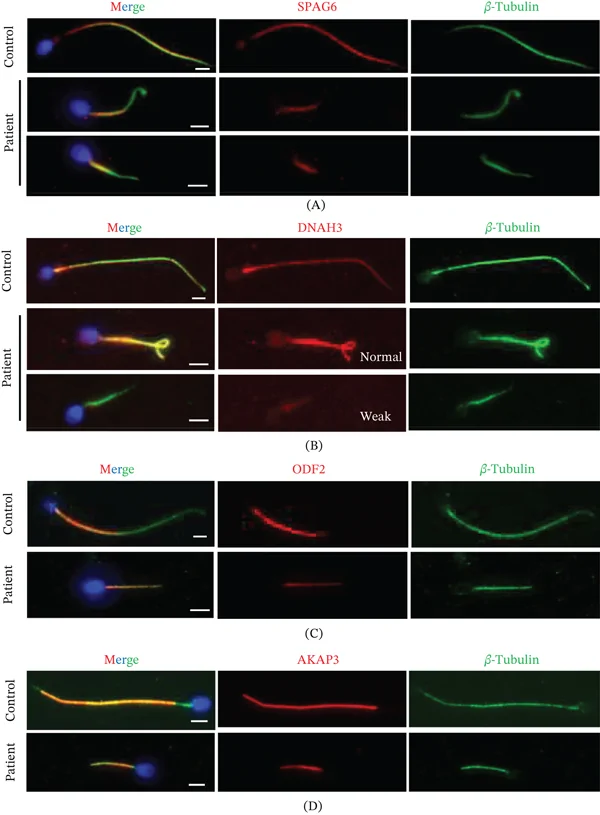
Immunofluorescence staining of spermatozoa from a control individual and the proband (IV:1) using antibodies against flagellar proteins and *β*‐tubulin. (A–D) Representative images of spermatozoa from a fertile normal control and the patient costained for *β*‐tubulin and (A) SPAG6, (B) DNAH3, (C) ODF2, or (D) AKAP3. Scale bars: 5 *μ*m.

To further characterize the ultrastructural defects, IF staining for SPAG6 (a CP marker), DNAH3 (an inner dynein arm [IDA] marker), ODF2 (an ODF marker), AKAP3 (a fibrous sheath [FS] marker), and *α*‐tubulin (a microtubule marker) was performed on spermatozoa from the patient and fertile controls. In control sperm, SPAG6 and DNAH3 were localized along the entire flagellum, whereas ODF2 was confined to the midpiece and principal piece, and AKAP3 was restricted to the principal piece, which was consistent with the known architecture of the flagellum (Figure 6A–D). In the patient′s sperm, DNAH3 signals were normal in 65.6% of the spermatozoa that were examined, and 34.4% displayed weak DNAH3 signals, whereas SPAG6 staining was partially absent along the flagellum, indicating CP deficiency. Notably, both ODF2 and AKAP3 signals extended along the entire length of the flagellum, suggesting the absence of the distal principal piece and the entire end piece. These results indicate that *ARL2BP* deficiency impairs axonemal organization, contributing to the MMAF in the patient.

## 4. Discussion

In the present study, a novel homozygous splice‐site mutation (c.294‐2A>G) in *ARL2BP* was identified in a Chinese male patient presenting with PCD and MMAF. The patient exhibited typical manifestations of PCD, including dextrocardia and bronchial cystic dilatation, along with severe OAT characterized by the absent, short, coiled, bent, and irregular flagella. Molecular analyses showed that the mutation induced aberrant splicing, resulting in nonsense‐mediated mRNA decay and the complete absence of ARL2BP protein expression. TEM and IF analyses demonstrated severe ultrastructural defects of the axoneme. These findings support *ARL2BP* as a causative gene for male infertility associated with MMAF and broaden the phenotypic spectrum of *ARL2BP*‐related ciliopathies.

ARL2BP localizes to the distal region of the connecting cilium and the periciliary extension of the inner segment, where it plays a role in ciliary doublet formation and axoneme elongation. Since the initial report by Davidson et al. [37], the association between *ARL2BP* mutations and ciliopathy phenotypes has been increasingly characterized. The classic triad of *ARL2B*P‐related disorders encompasses RP, situs inversus, and male infertility [40]. Moye et al. [40] reported patients with homozygous *ARL2BP* mutations presenting with RP and severe asthenozoospermia. Their spermatozoa displayed shortened and misassembled sperm tails, as well as the loss of axonemal doublets, findings comparable with those in our study. Since then, additional case reports have broadened the clinical manifestations associated with ARL2BP mutations. Zhu et al. [41] identified a patient with the variant c.22_23delAG presenting with RP, complete situs inversus, and oligozoospermia, whereas Placidi et al. [42] reported a case with rod‐cone dystrophy, situs inversus, asthenozoospermia, and unilateral renal agenesis with microcysts. Our patient exhibited overlapping characteristics with previously reported cases, including situs inversus and male infertility. Previous reports of ARL2BP‐related infertility mainly focused on basic semen parameters. In the present case, ultrastructural and IF analyses confirmed MMAF as the specific cause of spermatogenic failure, with spermatozoa showing characteristic defects such as partial absence of the CP microtubules, disorganization of peripheral doublets, and supernumerary ODFs. As reported for pathogenic variants in *DNAH1*, *DNAH2*, and *CCDC39*/*40*, *ARL2BP* deficiency causes severe disruption of the 9 + 2 axonemal architecture. However, several features appear particularly characteristic of *ARL2BP* loss‐of‐function. First, the consistent depletion of CP microtubules with SPAG6 deficiency, combined with peripheral doublet disarray, resembles the phenotype reported in *Arl2bp*‐knockout mice, in which no detectable doublet microtubules were observed. In contrast, *DNAH1* mutations typically cause loss of IDAs with relatively preserved CP structure, whereas *CCDC39*/*40* mutations primarily affect N‐DRC and radial spokes. Second, the presence of supernumerary ODFs and their disorganized distribution along the flagellum appear more prominent in ARL2BP deficiency, reflecting its specific role in microtubule doublet stabilization and accessory structure assembly. Third, the co‐occurrence of PCD and MMAF distinguishes *ARL2BP* from genes such as *DNAH1* or *CFAP43*/*44*, which typically cause isolated MMAF without respiratory manifestations. However, this case is remarkable for the absence of retinal involvement at presentation (Figure S1). Specifically, the fundus photographs revealed no bone spicule pigmentation or attenuated retinal vessels. Fundus autofluorescence showed no characteristic hyperautofluorescent ring or hypofluorescent patches. OCT demonstrated preserved retinal layer architecture with intact ellipsoid zone and outer nuclear layer. This difference may be attributed to the patient′s age (23 years), as RP in *ARL2BP*‐related disorders generally manifests during the third and fourth decades [37, 40, 41]. The absence of visual symptoms in our patient implies the possibility of age‐dependent penetrance or variable expressivity of retinal disease, highlighting the necessity for longitudinal ophthalmological follow‐up.

The phenotypic heterogeneity detected in patients with *ARL2BP* mutations suggests the significance of comprehensive clinical management. In terms of fertility, *ARL2BP*‐associated MMAF prevents natural conception, limiting reproductive alternatives to assisted reproductive technologies. The severe flagellar aberrations and immotility in patients with *ARL2BP* deficiency make intracytoplasmic sperm injection (ICSI) the principal treatment option. For retinal complications, previous studies have shown that RP symptoms generally manifest in the third to fourth decade of life, with nyctalopia and visual field constriction progressing over subsequent decades [37, 40]. Given the patient′s young age and the relatively well‐preserved central vision reported in the early stage of ARL2BP‐related RP, longitudinal ophthalmological follow‐up is necessary to detect potential delayed‐onset retinal degeneration.

In summary, this study identifies a novel splice‐site mutation in *ARL2BP* that is associated with a syndromic ciliopathy manifested as PCD and MMAF. We provide comprehensive ultrastructural and IF characterization of *ARL2BP*‐deficient sperm, specifically documenting consistent CP microtubule depletion and peripheral doublet disarray, features not previously detailed in human *ARL2BP* cases. These results broaden the recognized spectrum of *ARL2BP*‐related disorders, illustrate its role in preserving sperm flagellar axoneme integrity, and highlight the significance of genetic diagnosis in clinical management.

## Author Contributions

M.L., S.B., and X.J. conceived of the study, and participated in its design and coordination and helped to draft the manuscript. M.L., W.T., and D.Y. performed the experiments. Q.J., C.Z., Q.Z., and P.J. recruited the patient and performed semen analysis. J.W. and B.X. revised the manuscript of the article. M.L. and W.T. contributed equally to this work and share co‐first authorship.

## Funding

This study was supported by the 2024 Clinical Research Project of Anhui University of Chinese Medicine (2024YFYLCZX31); the China Postdoctoral Science Foundation (No. 2024M763169); Foundation for Innovative Research Groups of the National Natural Science Foundation of China (10.13039/501100001809; Nos. 82471657 and 82471657); the Health Research Program of Anhui (AHWJ2024Aa20579); and the Fundamental Research Funds for the Central Universities (WK9100000084).

## Disclosure

All authors read and approved the final manuscript.

## Conflicts of Interest

The authors declare no conflicts of interest.

## Supporting Information

Additional supporting information can be found online in the Supporting Information section.

## Supporting information


**Supporting Information 1** Figure S1: Clinical features images of the patient′s eye examination. The top and the bottom panels show findings from the left and right eyes, respectively, at 23 years of age. (i) Color fundus photographs; (ii) fundus autofluorescence images; and (iii) optical coherence tomography images.

**Supporting Information 2** Table S1: Overview of *ARL2BP* variant. The allele frequency of the *ARL2BP* mutant in the 1000 Genomes, ESP6500, ExAC, or gnomAD databases.

## Data Availability

The data that support the findings of this study are available in the supporting information of this article.
