# Supplementary material for: A Novel Homozygous Mutation in ARL2BP Causes Multiple Morphological Abnormalities of the Flagella and Primary Ciliary Dyskinesia
Source: Hum Mutat. 2026 Aug 25;2026:7635300. doi: 10.1155/humu/7635300 (PMC13504459; doi:10.1155/humu/7635300)
Supplement: Supplementary file 1 [file HUMU-2026-7635300-s002.docx]

| **Subjects** | **Genomic Position on chr16 (bp)** | **cDNA Change** | **Genotype** | **Allele Frequency in Population** | | |
| --- | --- | --- | --- | --- | --- | --- |
|  |  |  |  | **1KGP** | **ESP6500** | **gnomAD** |
| IV:1 | 57250410 | c.294-1G>C | splice acceptor | 0 | 0 | 1.239 × 10⁻⁶ |

**Table S1. Overview of *ARL2BP* variant**
